# Antiviral Efficacy of *Coridothymus capitatus* Essential Oil Against HSV-1 and HSV-2

**DOI:** 10.3390/life14081023

**Published:** 2024-08-18

**Authors:** Virginia Fuochi, Pio Maria Furneri, Salvatore Furnari, Adriana Garozzo

**Affiliations:** Dipartimento di Scienze Biomediche e Biotecnologiche (BIOMETEC), Università di Catania, 95030 Catania, Italy; furneri@unict.it (P.M.F.); salvatore.furnari2001@gmail.com (S.F.); agar@unict.it (A.G.)

**Keywords:** *Coridothymus capitatus*, essential oil, carvacrol, antiviral activity, HSV-1, HSV-2, natural antiviral agents, viral replication

## Abstract

*Coridothymus capitatus* is a perennial herb with aromatic leaves and flowers, distinct from *Thymus vulgaris* in its chemical composition, resulting in a unique Thymus Essential Oil (TEO). A main component of TEO, carvacrol, is known for its antimicrobial and insecticidal activity. Carvacrol has potent antibacterial, antioxidant, anti-inflammatory, and antifungal properties, generating interest in traditional medicine. However, studies on its antiviral activity are limited. Given the rise in viral infections and limitations of synthetic antiviral drugs, natural antiviral agents are promising due to their efficacy, lower resistance development, and reduced side effects. This study assessed the antiviral efficacy of TEO compared to that of pure carvacrol. We tested various viruses, revealing significant inhibitory effects of TEO on the replication of only *Simplexvirus humanalpha1* (HSV-1) and *Simplexvirus humanalpha2* (HSV-2), with specific interference during the early stages of the viral replication cycle after the adsorption period. TEO exhibited inhibitory effects at doses below the cytotoxic threshold, with IC_50_ values of 47 μg/mL for HSV-1 and 40 μg/mL for HSV-2. *Maximum* virus inhibition was achieved when TEO was added within 90 min post-infection, indicating interference with early viral replication steps. These findings highlight the potential of TEO as a natural antiviral agent and suggest further research into its mechanisms and clinical applications.

## 1. Introduction

*Coridothymus capitatus* (L.) Reichenb. fil. [syn. *Thymus capitatus* (L.) Hoffmanns. & Link], commonly known as Spanish Marjoram, is a perennial herb belonging to the *Lamiaceae* family [1]. Native to the Mediterranean region, it is widely cultivated for its aromatic leaves and flowers. While it shares similarities with *Thymus vulgaris* in terms of aromatic properties and traditional uses, there are notable differences in their chemical composition, contributing to distinct characteristics and potential applications [2,3]. One of the primary differences lies in the composition of their essential oils (EOs), which are volatile compounds obtained through the steam distillation of the plants’ leaves and flowers. *T. vulgaris* essential oil is predominantly composed of thymol, giving the plant its distinct aroma and contributing to its use as a culinary herb and natural remedy [4]. In contrast, essential oil from *Coridothymus*, hereinafter referred to simply as TEO (Thymus Essential Oil), contains major components such as carvacrol, β-caryophyllene, and p-cymene, known for their antifungal, antimicrobial, phytotoxic, and insecticidal activity [5]. Carvacrol, a monoterpene phenol, has been extensively studied for its antibacterial effects, exhibiting potent activity against a wide range of Gram-positive and Gram-negative bacteria, including antibiotic-resistant strains [6,7]. The antibacterial properties of carvacrol are attributed to its ability to disrupt bacterial cell membranes and inhibit essential enzymatic processes [6,8]. In addition to its antibacterial properties, TEO exhibits antioxidant, anti-inflammatory, and antifungal activities, generating interest in traditional medicine and alternative treatments. Despite these known properties, limited studies have been conducted on the antiviral activity of this essential oil [9,10,11].

The significance of finding natural antiviral agents has become increasingly important due to rising viral infections and the limitations of synthetic antiviral drugs. Natural antiviral agents offer a promising alternative because of their potential efficacy, lower likelihood of resistance development, and reduced side effects. *Coridothymus capitatus*, with its rich profile of bioactive compounds, stands out as a relevant candidate in this context. Exploring its essential oil could unveil new antiviral properties, contributing to the development of innovative and sustainable therapeutic options for combating viral infections [12,13,14]. This study aimed to assess the antiviral efficacy of TEO in comparison with that of pure carvacrol, reflecting the increasing interest in alternative approaches to combat viral infections.

## 2. Materials and Methods

### 2.1. Plant Material and Extract Preparation

Fresh *Coridothymus capitatus* (L.) Reichenb. fil. wild-harvested plants were collected during spring (May) from Val di Noto on the Iblei mountains in Sicily. The aerial parts of the plants were separated, thoroughly rinsed with water, and then air-dried at room temperature for 14 days. Essential oil (TEO, 100% pure) was extracted by steam distillation using 400 g of the plant to obtain 5 mL of pure essential oil (EXENTIAE SRL, L. 83118). 

Carvacrol 98% was supplied by SIGMA (Sigma-Aldrich® Merck KGaA, Darmstadt, Germania, Cod. 282197, ID PubChem: 24857025).

In the experimental procedures, TEO and carvacrol were initially dissolved in methanol to give a concentration of 94.3 and 97.6 mg/mL, respectively, and then, diluted in maintenance *medium* to achieve the final concentration needed. The dilution of the test compound contained a *maximum* concentration of 0.01% methanol, which was not toxic to the cell line used.

### 2.2. Viruses and Cells

*Simplexvirus humanalpha1* (Herpes simplex type 1, HSV-1: VR-260), *Simplexvirus humanalpha2* (Herpes simplex type 2, HSV-2: VR-734), and *Enterovirus betacoxsackie* (Coxsackievirus B1, VR-28) were propagated in VERO cells (CCL-81 ™); *Alphainfluenzavirus influenzae* (Influenza A virus, VR-1469) and *Betainfluenzavirus influenzae* (Influenza B virus, VR-1535) in were propagated in MDCK cells (NBL-2 CCL-34 ™); *Orthopneumovirus hominis* (Human respiratory syncytial virus, RSV: VR-1540), *Enterovirus coxsackiepol* (Poliovirus 1, VR-1562), and *Mastadenovirus caesari* (strain Human Adenovirus type 2, HAdV-2, VR-1080) were propagated in HEp2 cells (CCL-23 ™); and *Human Coronavirus OC-43*, which is actually included on genus *Alphacoronavirus*, subgenus *Duvinacovirus*, species *Alphacoronavirus chicagoense,* (https://ictv.global/taxonomy/taxondetails?taxnode_id=20083208, accessed on 12 August 2024, (OC43, VR-1558)) was propagated in HCT-8 cells ([HRT-18] CCL-244 ™). Viruses and cells were originally purchased from American Type Culture Collection (ATCC, LGC Standards S.r.l.–Italy Office, Sesto San Giovanni (MI) 20099, Italy). Cells were kept in a humidified 5% carbon dioxide atmosphere at 37 °C and grown in D-MEM (Dulbecco’s modified Eagle’s medium 30-2002™) supplemented with 10% heat-inactivated fetal bovine serum (FBS, 30-2020™), 200 μg/mL of streptomycin, and 200 units/mL of penicillin G (Penicillin-Streptomycin Solution 30-2300 ™). For the viruses tested, working stock solution was prepared as cellular lysates using *medium* with 2% FBS (maintenance *medium*).

### 2.3. Cell Viability

The cytotoxicity of the test compounds was evaluated by measuring their effect on cell morphology (e.g., rounding up, shrinking, detachment) via light microscopy after 24, 48, and 72 h. Cell growth was also determined via the 3-(4,5-dimethylthiazol-2-yl)-2,5-diphenyltetrazolium bromide (MTT) method, as previously described [15]. Briefly, cells were prepared in 96-well plates, and various concentrations of TEO were added. After 72 h of incubation at 37 °C, MTT (0.5 mg/mL) in *medium* without phenol red was added to each well. After a 3 h of incubation at 37 °C, the overlay was removed, and 100 µL of isopropanol was added to dissolve the blue crystals. The optical density (OD) was measured at 540 and 690 nm using a microplate reader (BioTek Synergy HTX Multimode Reader, Agilent Technologies, Inc., Santa Clara, CA 95051, USA) within 15 min of adding the isopropanol. The absorbance at 690 nm was subtracted from the absorbance at 540 nm to correct for non-specific absorption. Cell viability was expressed as a percentage of the untreated control values using the following formula:

Percentage=ODc−ODtODc∗100

where *ODc* and *ODt* indicate the absorbances of the untreated cell control and of the test sample, respectively. The 50% cytotoxic dose (CC_50_) calculated using dose–response curves and linear regression was expressed as the concentration of the compound that reduced the absorbance of the control sample by 50%.

### 2.4. Antiviral Activity

The antiviral activity of tested compounds was evaluated via a 50% plaque reduction assay, as previously described [15]. Briefly, confluent cells in 96-well plates were infected with 50 PFU of the virus/well. After 2 h of virus adsorption at 37 °C, an overlay medium with 1% methylcellulose, with or without test compounds at doses below the CC_50_, were added. After 24 h of incubation at 37 °C, plaques were visible in the virus controls. The overlay was then removed, and cells were stained with 1% crystal violet in methanol. Plaques were counted under a light microscope. Antiviral activity was calculated as the percentage reduction in plaque numbers, using the following formula:

$$Percentage=\frac{\left[n{}^{\circ}\;of\;plaques\;\left(control\right)-n{}^{\circ}\;of\;plaques\;\left(test\right)\right]}{n{}^{\circ}\;of\;plaques\;\left(control\right)}*100$$

The IC_50_, the concentration needed to reduce plaque formation by 50%, was determined using dose–response curves and linear regression. Mock-infected and infected cells without compounds served as cell and virus controls, respectively.

### 2.5. Cell Culture Pretreatment

Pretreatment of cultures was performed by exposing the cell monolayers to different concentrations of the compounds in maintenance *medium* for 2 h at 37 °C. After treatment, the cell monolayers were washed thoroughly with PBS and infected with HSV-1 and HSV-2 at an MOI (multiplicity of infection) of 0.1 to allow for viral cytopathic activity. The cell monolayers grown in maintenance *medium* without the test compounds were used as the control. Virus titration was performed as described above.

### 2.6. Virucidal Activity

Virucidal activity was performed as previously described by Zivna et al., 2024 [16]. To test possible virucidal activity, equal volumes (0.5 mL) of viral suspension and *medium* containing various concentration of the compounds were mixed and incubated for 2 h at 37 °C. Residual infectivity was determined via the plaque assay after dilution of the virus below the inhibitory concentration. Viruse-untreated controls were also included.

### 2.7. Effect of Time of Addition

The time of addition assay was performed as previously described [15]. Briefly, monolayers of cells were grown to confluence in 24-well plates and inoculated with viruses at an MOI of 0.1. The plates were incubated for 2 h at 4 °C to ensure synchronous replication of the viruses with or without compounds for the adsorption period. Then, the *inoculum* was removed, and *medium*, with or without the compound, was added at various times after the adsorption period (0–360 min). The plates were incubated at 37 °C for 8 h, cultures were then frozen, and virus yield was determined via plaque assay.

### 2.8. Statistical Analysis 

All experimental data are expressed as the mean standard error. Significance was assessed via ANOVA. *p* < 0.05 was considered to be statistically significant. Gompertz function was verified by R squared values >0.99 for all the data tested. 

## 3. Results

TEO has previously been subjected to chemical analysis in our earlier research [6]. The complete composition after continuous steam distillation is presented in Table 1. The predominant presence of carvacrol, comprising 73.04% of the oil, highlights its potential as the main bioactive component responsible for TEO’s antimicrobial and other biological activities. The total aromatic fraction constitutes 86.85% of the oil, further supporting its significant bioactive properties.

To assess the effect of the compounds on the growth and viability of human cellular lines, TEO and carvacrol were serially diluted and added to cell culture *medium*. The cytotoxic effect was characterized by the aggregation of degenerated cell clusters detaching from the bottom of the wells, leading to the formation of holes within the cell monolayer. The results detailing the cytotoxic effects of TEO and carvacrol are shown in Table 2. CC_50_ values equal to 470 µg/mL and 195 µg/mL were observed for TEO and carvacrol, respectively. 

**Figure 1:**
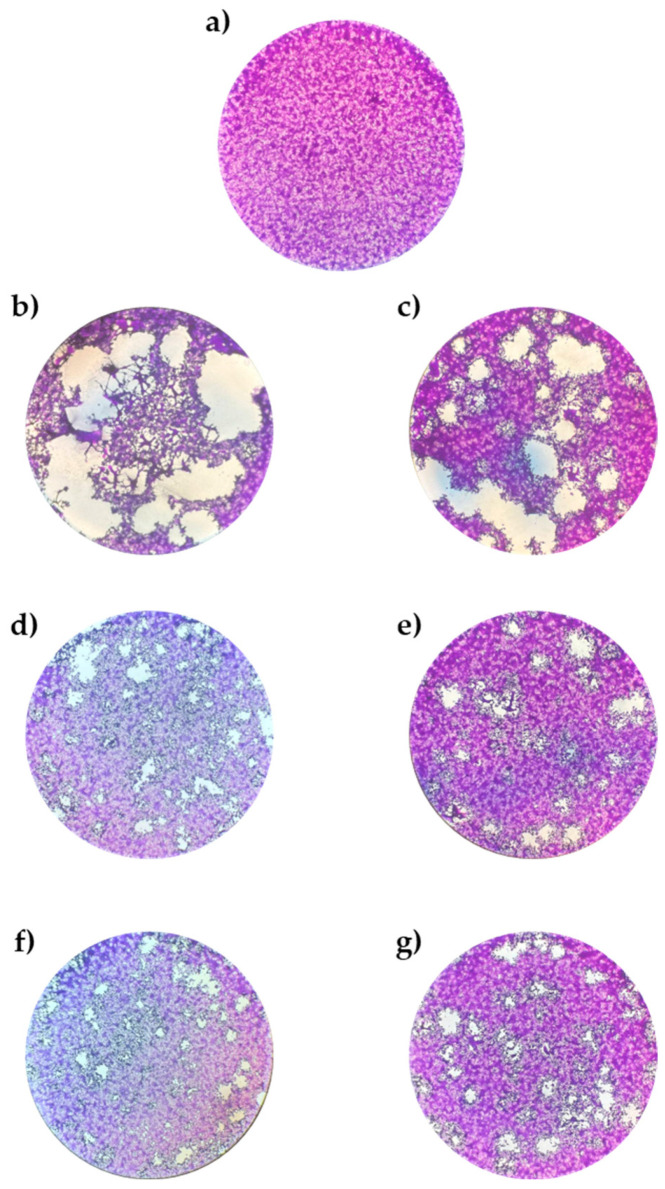
HSV-1 and HSV-2 morphology (100×); (**a**) VERO cells not infected as cell control; (**b**) HSV-1 plaques on VERO cells as virus control; (**c**) HSV-2 plaques on VERO cells as virus control; (**d**) HSV-1 plaques on VERO cells treated with TEO; (**e**) HSV-2 plaques on VERO cells treated with TEO; (**f**) HSV-1 plaques on VERO cells treated with carvacrol; (**g**) HSV-2 plaques on VERO cells treated with carvacrol. (TEO and carvacrol concentration equal to 100 µg/mL).

The findings revealed that TEO and carvacrol exhibited an inhibitory effect only against HSV-1 and HSV-2 (Figure 1). The IC_50_ values for TEO were determined to be 47 μg/mL and 40 μg/mL against HSV-1 and HSV-2, respectively. Carvacrol demonstrated comparable antiviral activity, with an IC_50_ of 49 μg/mL against both viruses (Table 3). Importantly, no activity was observed against the other viruses included in the testing panel. Since TEO showed activity against HSV-1 and HSV-2, we conducted further tests to understand its possible mechanism of action. Other viruses were excluded from subsequent analyses.

**Figure 2:**
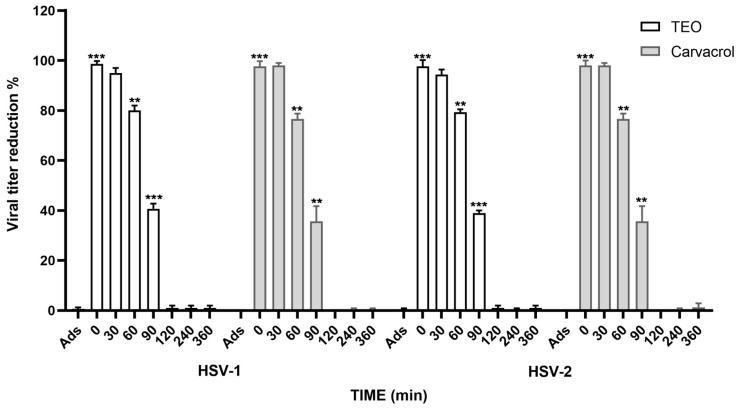
Results showing the maximal inhibition of the viruses if the compounds were added within 90 min of infection, after the adsorption period, indicating interference during an early step of the viral replicative cycle. ** *p* < 0.01; *** *p* < 0.001.

To unravel the mechanism behind the antiviral activity of TEO and carvacrol against HSV-1 and HSV-2, we conducted experiments to explore the mode of action. Specifically, we investigated whether these compounds inhibited the virus yield during a specific phase of the viral cycle by examining the time addition effect for HSV-1 and HSV-2. Results from these experiments unequivocally showed maximal virus inhibition when the compounds were added within 90 min of infection, post-adsorption. This suggests interference with an early step in the viral replicative cycle (Figure 2).

**Figure 3:**
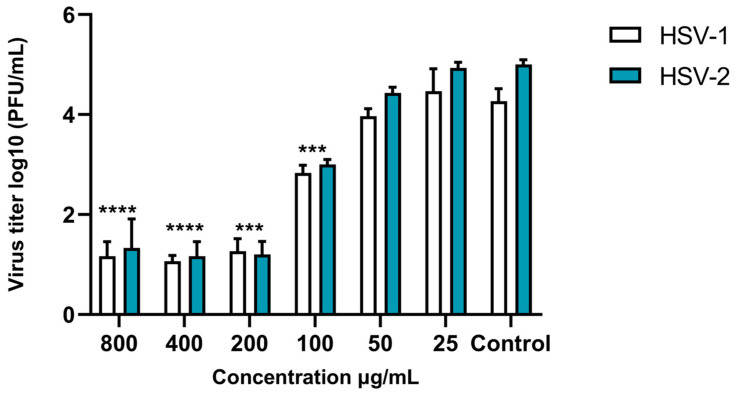
Dose-dependent virucidal activity of TEO against both HSV-1 and HSV-2 observed from 800 μg/mL to 25 μg/mL. Controls were tested with HSV-1 and HSV-2 to compare standard virus infections. (*** *p* < 0.001; **** *p* < 0.0001).

Given that the most sensitive period of the compounds coincided with an early event in HSV-1 replication, it was crucial to determine whether TEO and carvacrol exhibited a virucidal effect or protective action on VERO cells. No discernible effect was observed when cells were pre-treated with TEO or carvacrol. However, virucidal activity was demonstrated against both HSV-1 and HSV-2. The study of the compounds’ effect on neutralizing viral infectivity revealed a substantial reduction in viral titer (log10 PFU/mL) compared to that found with the virus control. Virucidal activity was found to be dose-dependent against HSV-1 and HSV-2 (Figure 3). The same effect was observed for carvacrol, with results comparable to those of TEO, and no statistically significant differences were found. Importantly, none of the tested compounds displayed virucidal activity against the other viruses examined.

## 4. Discussion

This research investigated the antiviral properties of *Coridothymus capitatus* essential oil and its main component, carvacrol, against a range of viruses. TEO, derived from the Spanish marjoram plant, is chemically distinct from *Thymus vulgaris* essential oil, which is predominantly composed of thymol [2,5,17,18,19]. Our previous study highlighted the predominance of carvacrol in TEO [6]. Carvacrol, a monoterpene phenol, is well known for its potent antibacterial effects, disrupting bacterial cell membranes and inhibiting crucial enzymatic processes. Numerous studies have demonstrated its effectiveness against various bacteria, including multi-resistant strains [8,20,21,22,23,24,25,26,27,28]. 

While *Thymus vulgaris* essential oil is known for its antiviral properties, TEO, with its high carvacrol content, offers a different chemical profile [10,29,30]. Therefore, this study aimed to investigate the antiviral potential of TEO by initially screening a broad panel of viruses, including polio 1, Adeno 2, Coxsackie B1, HSV-1, HSV-2, RSV, Influenza A and B, and Coronavirus OC43. After observing significant inhibitory effects specifically on the replication of HSV-1 and HSV-2 at concentrations much lower than the cytotoxic threshold, we decided to focus our subsequent investigations on these herpes viruses. This targeted approach allowed us to conduct more detailed experiments to better understand the mechanisms underlying TEO’s antiviral activity against HSV-1 and HSV-2. Moreover, TEO demonstrated slightly stronger antiviral activity than carvacrol when tested as a single component.

This research explored the mode of action of these compounds, revealing specificity during the early stages of the viral replication cycle, suggesting potential interference with early replication events after adsorption period. Additionally, virucidal activity was demonstrated against HSV-1 and HSV-2, indicating a reduction in viral infectivity, although this effect was not observed for other tested viruses.

These results contribute valuable insights into the multifaceted interactions between essential oils, particularly TEO, and viruses. The identified antiviral properties of TEO, especially against HSV-1 and HSV-2, open up avenues for further exploration and development of therapeutic interventions. The dose-dependent virucidal activity further emphasizes the potential applicability of TEO in combating viral infections.

This study provides valuable insights into the antiviral potential of TEO and its main component, carvacrol. However, several limitations need to be acknowledged. First, the study focused exclusively on in vitro experiments, which, while informative, do not fully replicate the complexity of viral infections in a living organism. Further in vivo studies are necessary to validate these findings and assess the therapeutic potential and safety profile of TEO in more realistic biological settings.

Although a broad panel of viruses with diverse characteristics was tested, the essential oil demonstrated inhibitory effects exclusively against HSV-1 and HSV-2. This specificity suggested that the components of the essential oil are particularly effective against the mechanisms involved in the replication or structural integrity of HSV-1 and HSV-2, as demonstrated by a virucidal experiment.

Additionally, the exact mechanism by which TEO and carvacrol exert their antiviral effects is not fully understood.

Prospective research should also focus on optimizing the formulation and delivery of TEO for clinical use. Investigating the stability, bioavailability, and pharmacokinetics of TEO in various delivery systems will be crucial for developing practical therapeutic applications. Furthermore, clinical trials are essential to confirm the efficacy and safety of TEO in human populations, which will be the ultimate test of its potential as a natural antiviral agent.

Therefore, this research supports the broader interest in alternative and natural approaches to healthcare [31,32,33,34]. The growing attention towards sustainable and natural healthcare solutions makes the exploration of essential oils, with their diverse bioactive compounds, a promising avenue for future therapeutic interventions.

## 5. Conclusions

In conclusion, our research explored the antiviral properties of *Coridothymus capitatus* essential oil and its main component, carvacrol, highlighting their distinctive chemical composition compared to *Thymus vulgaris*. This study demonstrated significant inhibitory effects on the replication of HSV-1 and HSV-2, with TEO showing greater potency than carvacrol alone. Notably, dose-dependent virucidal activity against these viruses was observed, indicating a reduction in viral infectivity.

Our results suggest that TEO specifically interferes with the early stages of the viral replication cycle, emphasizing its potential in combating viral infections. These findings provide valuable insights into the complex interactions between essential oils and viruses and present promising opportunities for developing therapeutic interventions.

Given the growing interest in alternative and natural healthcare approaches, our research underscores the potential of TEO in addressing viral infections. One practical application could be the incorporation of TEO into topical formulations, such as creams and ointments, to treat HSV infections. This approach leverages TEO’s potent antiviral and virucidal activities, providing a natural and effective treatment option for managing HSV outbreaks on the skin and mucous membranes.

However, further comprehensive research is needed to fully elucidate the antiviral properties of TEO and explore its potential applications in healthcare. Future studies should focus on detailed mechanism analyses, clinical trials, and investigating its efficacy against a broader range of viral infections. Additionally, the development of optimized topical formulations and delivery systems for TEO could enhance its therapeutic potential and user convenience, paving the way for its use in clinical settings.

## Figures and Tables

**Table 1:** Chemical composition of TEO ^a^.

Method of Extraction	Major Components	(%)
Continuous steam distillation	p-Cymene	9.48
	γ-Terpinene	4.33
	Carvacrol	73.04
	**Total aromatic fraction**	**86.85**
	β-Thujene	1.16
	α-Pinene	1.20
	β-Myrcene	1.37
	Terpinolene	1.70
	β-Caryophillene	5.05
	Terpenoid fraction	10.48
	**Total identified**	**97.33**

^a^ Data shown were provided by the manufacturer.

**Table 2:** Cytotoxic activity expressed as ^a^ CC_50_ (µg/mL) of the compounds on cellular lines.

Compounds	VERO	MDCK	HEp2	HCT8
TEO	470	56	113	94
Carvacrol	195	23	47	39

^a^ Values are mean ± 0.5 S.D. for three separate assays.

**Table 3:** Antiviral activity of the compounds against all viruses tested.

Virus	^a^ IC_50_ (µg/mL)	
	Carvacrol	TEO
HSV-1	49	47
HSV-2	49	40
Coxsackievirus B1	>195	>470
A influenza virus	>23	>56
B influenza virus	>23	>56
RSV	>47	>113
Polio 1	>47	>113
HAdV-2	>47	>113
Coronavirus OC43	>39	>94

^a^ Values are mean ± 0.5 S.D. for three separate assays.

## Data Availability

Data is contained within the article.
